# Evolution of antibody profiles against SARS-CoV-2 in experienced and naïve vaccinated elderly people

**DOI:** 10.3389/fimmu.2023.1128302

**Published:** 2023-02-22

**Authors:** Iván Sanz-Muñoz, Rosa López-Mongil, Javier Sánchez-Martínez, Laura Sánchez-de Prada, Marta Domínguez-Gil González, Diana Pérez-SanJose, Silvia Rojo-Rello, Cristina Hernán-García, Virginia Fernández-Espinilla, Raúl Ortiz de Lejarazu-Leonardo, Javier Castrodeza-Sanz, José María Eiros

**Affiliations:** ^1^ National Influenza Centre, Edificio Rondilla, Hospital Clínico Universitario de Valladolid, Valladolid, Spain; ^2^ Instituto de Estudios de Ciencias de la Salud de Castilla y León, (ICSCYL), Soria, Spain; ^3^ Dr. Villacian Nursing Home, Valladolid, Spain; ^4^ Microbiology Unit, Hospital Universitario Río Hortega de Valladolid, Valladolid, Spain; ^5^ Microbiology Unit, Hospital Clínico Universitario de Valladolid, Valladolid, Spain; ^6^ Preventive Medicine and Public Health Unit, Hospital Clínico Universitario de Valladolid, Valladolid, Spain

**Keywords:** COVID-19, SARS – CoV – 2, nursing homes, elderly, third dose (booster), booster

## Abstract

**Introduction:**

The third dose of the COVID-19 vaccine is especially necessary in people over 65 years of age due to their lower immune response.

**Methods:**

We designed a multicentre, prospective observational study including 98 people ≤65 years old who lived in two nursing homes in Valladolid, Spain. One of the groups had previous experience with SARS-CoV-2 (n=68;69.4%) and the other was naïve (n=30;30.6%). We evaluated the response to the three doses of the Comirnaty vaccine and the dynamics of antibodies during 5 consecutive serum samplings: 2 after the first two doses of vaccination, one three months after the first dose, another at 6 months and the last one month after the third dose. IgG antibodies against SARS-CoV-2 S1, RBD and N antigens were analysed.

**Results:**

Both groups increased the level of Abs against S1 and RBD, but the experienced group showed a 130-fold higher humoral response due to hybrid immunisation (infection+vaccination). The response to vaccination with Comirnaty against COVID-19 was higher in those ≤65 years with previous experience than those who were naïve. However, the amount of antibodies against S1 and RBD equalised at 6 months. After the third dose, both groups raised the amount of antibodies to a similar level. The reinfections suggested by the analysis of antibodies against N were frequent in both groups.

**Discussion:**

The third dose showed a clear benefit for elderly people, with the reinforcement of the antibody levels after the decline suffered after six months of the first two doses.

## Introduction

Since the beginning of the COVID-19 pandemic, more than 650 million cases and 6.5 million deaths caused by SARS-CoV-2 have been reported to the World Health Organization (WHO) (1). This new infectious disease has shown a particularly high burden of hospitalisation and mortality in people over 50 years of age, especially in those over 70 years of age (2).

Nursing home residents account for a disproportionally higher mortality rate compared with their same-age community counterparts (3–7). For example, in the United States, the deaths of people living in nursing homes account for 40% of the total, which demonstrates the vulnerability of these facilities to rapidly transmitted infectious diseases, such as COVID-19 (8–10).

For many infectious diseases, vaccination is the most effective way to limit the spread of the disease and the severity of cases (11–13). This practice has been essential for limiting the COVID-19 pandemic, and in many Western countries, mortality has fallen in all age groups (14, 15), especially in the populations at risk, since the beginning of vaccination.

In Spain, a massive vaccination campaign started at the end of 2020, with the highest priority given to nursing home residents (16). Vaccines reduce the risk of severe and complicated COVID-19 infection (17, 18). However, the susceptibility of the institutionalised population makes the follow-up and evaluation of humoral responses of special importance to update boosters and vaccine programmes when appropriate and to avoid outbreaks and fatal consequences. It is important to determine how the antibodies (Abs) elicited by the COVID-19 vaccine evolve, how booster doses increase them, and to what extent they can protect the elderly population.

In this study, we assessed the dynamics of Abs against three different SARS-CoV-2 epitopes after three vaccination doses (complete dosage and booster) in two cohorts of nursing home residents: those who had previous experience (EX) with COVID-19 after the first wave and naïve (NA) residents.

## Materials and methods

### Study design

**Figure 1:**
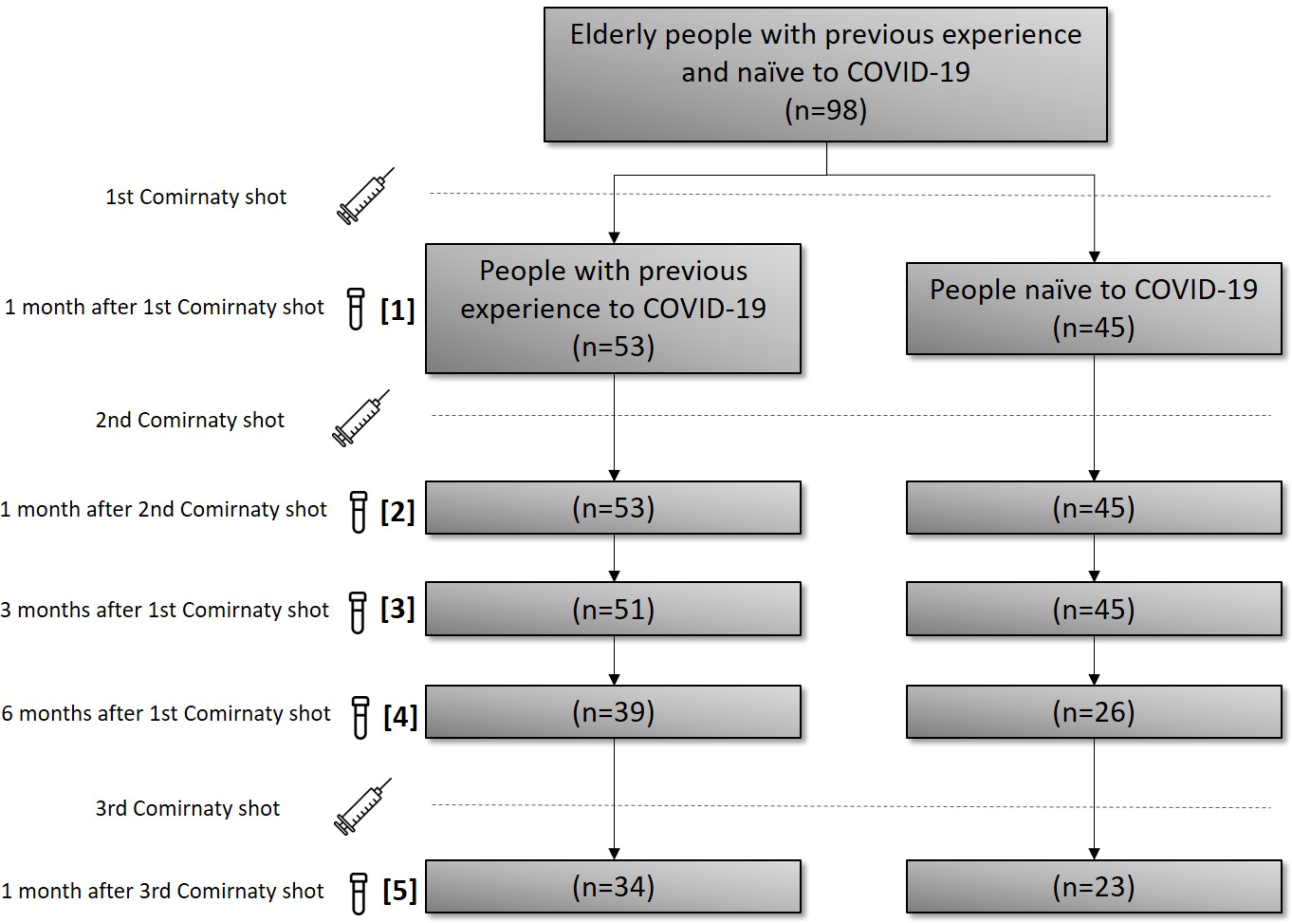
CONSORT diagram for patient selection, sampling dates and allocation. In brackets, the consecutive sampling number is described for guidance.

A quasi-experimental, case–control, uncontrolled before–after, prospective, and analytical multicentre study was carried out in two public nursing homes in the city of Valladolid, Spain. This study was conducted between January 2021 and January 2022. The inclusion criterion was as follows: residents ≥65 years that received the three doses of the Comirnaty vaccine (Pfizer-BioNTech) (first two doses and a monovalent booster) and that agreed to participate in the study by signing an informed consent. In this study, 98 elderly subjects of 350 residents (28.0%) were included. Recruitment included all people who wanted to participate in both nursing homes. The 98 recruited persons were classified into two different groups depending on their previous exposure to SARS-CoV-2. The first group (cases) comprised 68 individuals (69.4%) with previous experience (EX) with COVID-19 (previously diagnosed by clinical diagnosis, PCR, antigen tests, or the antibodies test against N protein used in our work), and the second group (controls) comprised 30 NA individuals (30.6%) with SARS-CoV-2 infection. These people received two doses of the Comirnaty vaccine and the monovalent booster of the same vaccine (**Figure 1**).

The serum samples were collected in both groups at the following times: 1 month after the first dose of vaccine, 1 month after the second dose, 3 months after the first dose, and 6 months after the first dose. An additional sample was collected 1 month after the third/booster dose. Overall, 21 persons in the EX group and 8 in the NA group abandoned the study (drop-out), and 5 persons in the EX group and 7 in the NA group died. The death was caused by COVID-19 in only 3 of 12 persons that died during the study, one in the EX group and 2 in the NA group. The other deaths were triggered by other causes.

Both nursing homes were monitored prior to the administration of the vaccine and since the beginning of the pandemic in March 2020, so COVID-19 infection records were known by either clinical diagnosis or laboratory confirmation by PCR or antigen testing. This study was approved by the Ethics Committee of the Eastern Health Area of Valladolid (code: PI 21-2101). Written informed consent was obtained before sampling. This research was carried out according to the Declaration of Helsinki.

### Humoral response evaluation

An analysis of IgG Abs against three SARS-CoV-2 antigens was performed using the 3-plex S1-N-RBD for SARS-CoV-2-IgG reagents (Luminex, Austin, TX, USA). This analysis relatively quantifies the presence of specific IgG antibodies against the S1 domain, the receptor-binding domain (RBD) of the S protein (antigen included in the COVID-19 vaccine), and the N protein. This detection was performed in arbitrary units (AU/µl). The detection assay is a modified ELISA (enzyme-linked immunosorbent assay) where Abs is specifically coupled to magnetic microspheres, and by means of a specific biotinylated secondary antibody against S1, RBD, and N IgG, those Abs are detected and quantified by the Luminex 100 system (Luminex). To determine the minimum–maximum limit of the initial dilution of this technique, several dilutions of positive serum samples and controls were performed. Thus, 1/8,000 was determined as the best dilution. A positive control and a negative control were included in duplicate for each assay. The negative control (included in the reagents) consisted of pre-pandemic sera negative for SARS-CoV-2 and other endemic human coronaviruses. However, the positive control (included also in the reagents) consisted of a pool of positive serum samples for SARS-CoV-2. The cutoff of the technique was established following the manufacturer’s specifications, establishing a positive threshold of 2000 AU/µl.

### Statistical analysis

To analyse the serological data, the AU/µl were multiplied by 20 to obtain a result equivalent to the original dilution indicated in the protocol. The statistical analysis was carried out by comparing the AU/µl values between both elderly groups. Those AU/µl were analysed using the median and IQR (interquartile range). The results were analysed in a Mann–Whitney test to compare the Abs values among groups and the mean ages, Student’s T to compare the age among groups, and a chi-square test to compare the proportion of men to women, using SPSS v 27 (IBM) and Prism GraphPad v7.

## Results

### Population characteristics

The mean age was 83.0 years (CI95%, 81.0–85.1) for the EX group and 80.1 years (CI95%, 76.6–83.6) for the NA group, with no significant differences among them (p > 0.05). The percentage of men was 44.1% in the EX group and 36.7% in the NA group, with no significant differences (p > 0.05). The three persons who died with COVID-19 were two men 71 and 88 years old and one woman 96 years old. The 71-year-old man died before the sampling at 6 months after the first dose, and the other two died before being vaccinated with the third dose.

### Experienced and naïve elderly people show different antibody profiles at the beginning but had similar profiles after the booster

After the first dose of vaccine, all subjects in the EX group had detectable antibodies (≥2,000 AU/µl) against the S1 and RBD antigens, except for two men aged 94 and 95 years and a 93-year-old woman, who showed Abs levels above this threshold after the second dose. In the NA group, 17 individuals (56.7%) showed detectable antibodies against S1 and 22 (73.3%) against RBD after the first dose. After the second vaccine dose, all individuals in both groups showed detectable antibodies against both antigens.

**Figure 2:**
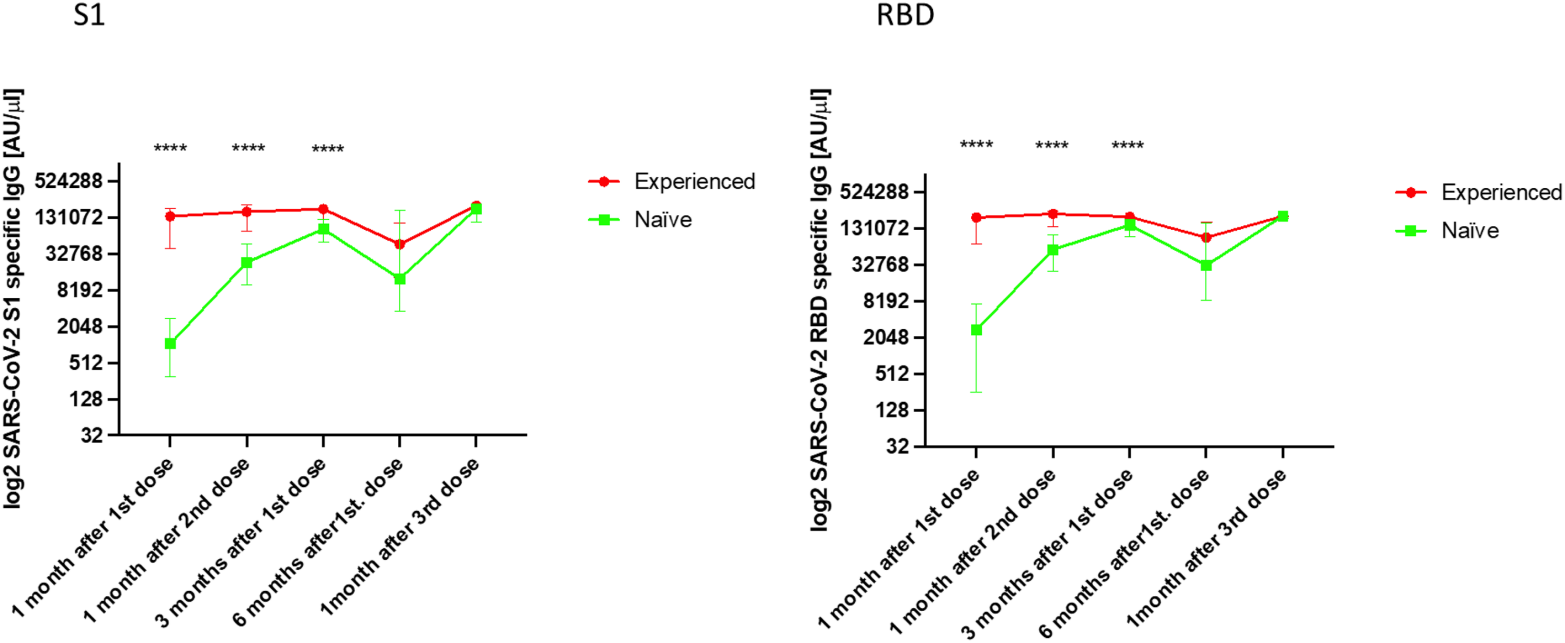
Median IgG levels [log2 AU/µl] (arbitrary units/µl [IQR]) against the S1 and RBD antigens of SARS-CoV-2 in both the experienced and naïve elderly groups analysed. ****p < 0.0001.

First, we analysed the dynamics of IgG Abs against S1 and RBD in the five samples taken during the study. After the first two vaccine doses, both groups had an increased Abs level against S1 and RBD until the third month after the first dose. The highest median Abs levels against S1 were observed for both the EX and NA groups in the sampling after the booster dose (sampling 5) (EX: 206,348 [IQR, 157,774–209,216]) (NA: 184,504 [IQR, 108,848–207,672]) (**Supplementary file 1** and **Figure 2**). However, the highest median levels of Abs against the RBD antigen were observed 1 month after the second dose in the EX group (228,936 [IQR, 142,293–249,242]), whereas in the NA group, it was observed after the booster dose (sampling 5) (211,360 [IQR, 190,708–214,232]).

The median Abs levels were significantly higher in the first three samples in the EX group compared with the NA group (p < 0.0001) against both S1 and RBD. In fact, these Abs levels were 130-fold higher after the first dose, 6.9-fold higher after the second dose, and 2.1-fold higher 3 months after the second dose in the EX group than in the NA group. Six months after the first vaccine dose, the median Abs levels against S1 and RBD decreased in both groups to a similar level, without a significant difference between them (p > 0.05).

One month after the administration of the booster dose, an increase in the median Abs levels against S1 and RBD was observed in both groups. This increase was similar, and significant differences were not found between the Abs levels obtained by either group against both vaccine antigens (p > 0.05).

### Exposure to SARS-CoV-2 after vaccination

**Figure 3:**
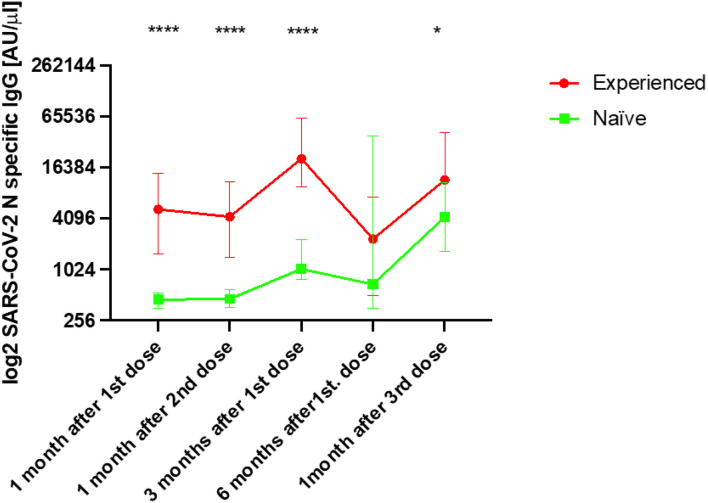
Median IgG levels [log2 AU/µl] (arbitrary units/µl [IQR]) against the S1 and RBD antigens of SARS-CoV-2 in both the experienced and naïve elderly groups analysed. *p < 0.05, ****p < 0.0001.

We analysed the dynamics of Abs against the N antigen, which shows natural exposure to the virus. The median values of Abs against the N protein observed during the first three samplings were significantly higher in the EX group than in the NA group (p < 0.001) (**Table 1** and **Figure 3**). After the fourth sampling, the values fell but continued to be higher in EX than in NA but without significance. After the third dose, we observed that the EX group showed significantly higher median values of Abs against the N antigen than the NA group (p < 0.05).

**Table 1:** Median IgG (AU/µl [IQR]) levels in each sampling against the N antigen of SARS-CoV-2 in the group that had previous experience with the virus (experienced) and the group that was not previously infected (naïve).

	N antigen [median AU/μl (IQR)]		
Sampling	Experienced	Naïve	p-value
1 month after the 1st dose	5,306 (1,554–14,129)	454 (356–546)	<0.0001
1 month after the 2nd dose	4,316 (1,443–11,306)	462 (365–593)	<0.0001
3 months after the 1st dose	20,972 (9,668–63,500)	1,042 (784–2,318)	<0.0001
6 months after the 1st dose	2,364 (510–7,403)	688 (356–38,916)	0.8683
1 month after the 3rd dose	11,768 (3,938–42,494)	4,312 (1,696–10,968)	0.0304

Threshold for negative results <2,000 AU/µl.

The data showed that the EX group had positive Abs levels during the first two samplings, and those dramatically increased 3 months after the first dose, indicating that this group had intense contact with the virus before this sampling. Specifically, a total of 58 (87.9%) persons presented a ≥2-fold induction of initial Abs against N with respect to the previous sampling. We also observed that a total of 30 persons (73.2%) had an increased Abs against the N antigen between the sampling 6 months after the first dose (fourth sampling) and after the third dose of the vaccine. In the NA group, we also observed a ≥2-fold induction increase of Abs against the N protein 3 months after the first dose in 20 persons (66.7%), but this increase was higher after 6 months since the first dose included seven more persons with Abs against the N protein.

**Figure 4:**
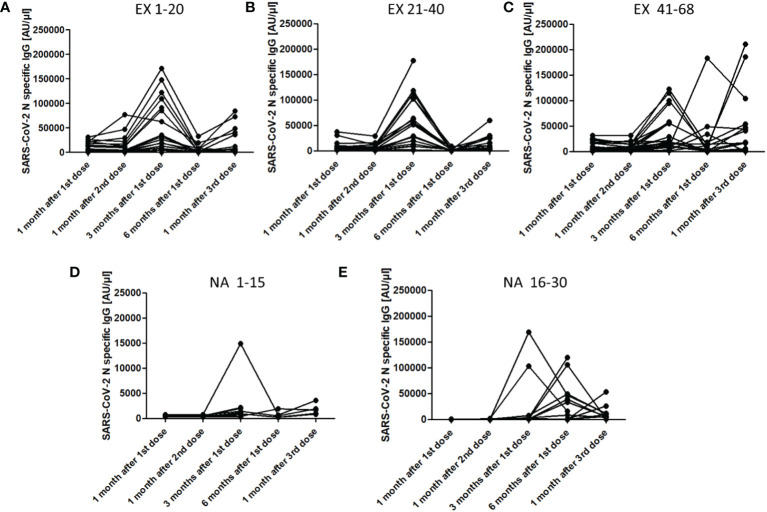
IgG levels [AU/µl] (arbitrary units/µl) against the N antigen of SARS-CoV-2 in each person included in both the experienced and naïve elderly groups. **(A)**, Experienced individuals from 1 to 20; **(B)**, Experienced individuals from 21 to 40; **(C)**, Experienced individuals from 41 to 68; **(D)**, Naïve individuals from 1 to 15; **(E)**, Naïve individuals from 16 to 30. The number indicates the individual position of each person in the study.

When we analysed the evolution of the antibodies against the N protein in each individual of both groups, we observed that the majority of the individuals in the EX group had increased values 3 months after the first dose (**Figure 4**). Many of them also showed an increase but of a lesser magnitude 1 month after the third dose. In the case of the NA group, this increase occurred mostly 6 months after the first dose, except for three individuals who had increased antibodies against the N protein 3 months after the first dose. Of those three patients that died due to COVID-19, one of them died after the third sampling, in which a 12.7-fold increase in N Abs was observed, and the other died 3 months after suffering a 55.7-fold increase in these Abs levels. The S1 and RBD Abs levels of two of the three persons who died due to COVID-19 were lower in all samples from the first sampling until death. For example, in the case of patient EX60, the S1 and RBD IgG levels were 1,268 and 2,816 AU/µl, respectively, in sampling 1, 4,524 and 10,984 AU/µl, respectively, in sampling 2, and 29,952 and 80,092 AU/µl, respectively, in sampling 3, which were the last values recorded before death. All values were lower than the mean IgG levels detected in the EX group. In the second subject that died (subject NA18), the S1 and RBD IgG levels were 1,192 and 3,396 AU/µl, respectively, in sampling 1, 2,768 and 7,056 AU/µl, respectively, in sampling 2, 4,672 and 34,172, respectively, in sampling 3, and 760 and 1,596 AU/µl, respectively, in sampling 4, which was the last sampling before death. The third subject (subject NA27) died with COVID-19 but had IgG levels above the median of the NA group.

## Discussion

The data from our study show that the response to vaccination with the Comirnaty vaccine was greater in those individuals ≥65 years who were previously infected with SARS-CoV-2 compared with those who were naïve. After the first dose of the vaccine, the humoral response was 53-fold higher in EX than in NA, and after the second dose, it was only 4.5-fold higher. This demonstrates that hybrid immunisation (infection+immunisation) provides a higher intensity response of Abs against the vaccine antigens than in those elderly who were only vaccinated. Our data corroborate previous works (19–25), highlighting the role of hybrid immunity (20, 26–28).

The level of Abs and its dynamics tend to overlap between both groups as time progresses since vaccination, despite their initial differences. In both groups, the highest level of Abs was observed 2–3 months after the first vaccine dose, followed by a sharp drop after the sixth month, which has also been observed by other authors (29). This fall was so intense in the EX group that the Abs levels against the S1 antigen in the NA were even higher after the third sampling than in EX, despite that, previously, they were always lower. This is important because although hybrid immunity induces higher Abs responses after vaccination in the elderly, after 6 months, the Abs levels seem to be the same in EX compared with NA.

After the booster dose, the EX group recovered the Abs level to a similar extent to that observed after the second dose, whereas the NA group increased to similar values compared with those observed in the EX group, two times higher than the NA group obtained after the second dose. This confirms that a third dose in people ≥65 years old is necessary for recovering Abs levels after 1 year. Other authors have observed similar results, with a powerful booster effect that helps strengthen immunity, not only in patients over 65 years of age but also in other risk groups (30, 31).

The analysis of the Abs against the N protein showed that, after vaccination, exposure to SARS-CoV-2 may be frequent in both the EX and NA groups. As the N protein is not an antigen included in the vaccine, their increase can only be possible if the virus infects the people. However, we were not able to confirm that infection using PCR or sequencing, so this was a weakness of our work. Our data showed that these new exposures to the virus occurred earlier in those people who had hybrid immunity than in those without a previous contact with SARS-CoV-2. This was surprising because some authors have demonstrated that hybrid immunity presents a more durable response (32), in contrast with the results of our work. However, we think that this behaviour could be a biased interpretation because people with hybrid immunity could be overreacting to SARS-CoV-2 exposure due to previous experience with the virus, increasing their Abs faster and higher against the N protein. In contrast, in the NA group that had no previous experience with the virus, their first natural contact with the N protein seemed not to increase the Abs levels as much as in the EX group. Further research is needed to investigate this particularity.

Some studies have shown that reinfections in nursing homes have been frequent during the pandemic, being more likely than in young people and adults (33). These same authors showed that the mean time to reinfection was 135 days (33), which is consistent with the decrease in protection observed in our study 6 months after the first vaccine dose. This makes residences one of the sites with the highest risk for reinfections, probably due to the socio-sanitary conditions of these centres (34).

However, having suffered reinfections did not seem to have significantly affected COVID-19-specific mortality since only three people of the 98 who began the study died from this during the study, two of whom suffered a 12- and 57-fold increase in the N Abs values before dying. Other researchers have seen an association between reinfections and the risk of dying in the elderly (35). However, this type of study performs recurrent PCR tests, so it does not evaluate the humoral response that can show contact with the virus that is not visible with molecular diagnosis, as in our work. Our data showed that reinfections occur in people older than 65 years and that, with the convenient protection derived from vaccination, it could be less harmful than in naïve people.

The main limitation of this work is that since we have not performed constant and recurrent PCR or antigen tests to detect SARS-CoV-2 infection in patients that have increased N Abs levels, we cannot guarantee that they have become infected between the samplings described above. However, the loss of individuals due to all-cause mortality or because they withdrew from participating in the study significantly reduced the number of people participating in the last two samplings. Additionally, more sampling could offer valuable information about the behaviour of antibodies during the study. It was not possible to perform a complementary analysis using the WHO international standard for anti-SARS-CoV-2 immunoglobulin; therefore, we do not know the level of standardised protection that the Abs observed could imply. In addition, since the technique used was a modified ELISA detecting total Abs against S1, RBD, and N, we do not know which of them may be the neutralising type Abs; thus, these results cannot be compared with Plaque Reduction Neutralisation Tests (PRNT).

## Conclusions

In conclusion, the response to vaccination with the Comirnaty vaccine against COVID-19 was higher in those ≥65 years old who had previous experience with SARS-CoV-2 compared with those naïve elderly people. After 6 months, a general decrease in Abs was observed in both groups, leading to a similar amount of Abs among the groups. However, the monovalent booster increased the level of antibodies up to the values observed after the second dose, inducing good reinforcement of Abs levels after 1 year. The reinfections assessed only by the immune response in the elderly cohorts studied were frequent, being later in the naïve group than in the experienced one.

## Data availability statement

The raw data supporting the conclusions of this article will be made available by the authors, without undue reservation.

## Ethics statement

The studies involving human participants were reviewed and approved by Ethics Committee of the Eastern Health Area of Valladolid. The patients/participants provided their written informed consent to participate in this study.

## Author contributions

IS-M, RL-M, RL-L, JC-S, and JE designed the study. RL-M recruited the samples. IS-M, JS-M, LS-d, MG, DP-S, and SR-R performed the experiments. IS-M, RL-M, LS-d, SR-R, MG, CH-G, and VF-E wrote the manuscript. IS-M, RL-M, RL-L, JC-S, and JE revised the manuscript. All authors contributed to the article and approved the submitted version.

## Supplementary material

Supplementary file 1 Median IgG (AU/µl [IQR]) levels in each sampling against S1 and RBD antigens of SARS-CoV-2 in the group that had previous experience with the virus (experienced) and the group that was not previously infected (naïve). Threshold for negative results <2000 AU/µl.
